# Epidemiology A Metabolite Score Explains Relationships of Unintentional Weight Loss with Mortality and Mobility Limitation

**DOI:** 10.1093/geroni/igaf122.2959

**Published:** 2025-12-31

**Authors:** Shanshan Yao, Megan Marron, Samaneh Farsijani, Iva Miljkovic, George Tseng, Ravi Shah, Venkatesh Murthy, Anne Newman

**Affiliations:** University of Michigan, Ann Arbor, Michigan, United States; University of Pittsburgh, Pittsburgh, Pennsylvania, United States; University of Pittsburgh, Pittsburgh, Pennsylvania, United States; University of Pittsburgh, Pittsburgh, Pennsylvania, United States; University of Pittsburgh, Pittsburgh, Pennsylvania, United States; Vanderbilt University Medical Center, Nashville, Tennessee, United States; University of Michigan, Ann Arbor, Michigan, United States; University of Pittsburgh, Pittsburgh, Pennsylvania, United States

## Abstract

Unintentional weight loss (UWL) is related to mortality and functional decline. Using 77 metabolites associated with UWL risk in the Health, Aging and Body Composition participants, we investigated whether a composite metabolite score explained the relationship of UWL with mortality and mobility limitation. First, we selected 27 metabolites associated with incident UWL (>3% annual loss vs weight stable) using penalized logistic regression. Next, we created an UWL metabolite score, sum of 27 standardized metabolites weighted by their association with incident UWL, with a higher score indicating a greater potential risk for UWL. Among 2,286 participants (mean age 75, 37% Black, 51% women), those with UWL had 60% higher mortality and 25% higher hazard of mobility limitation risk after weight loss compared with weight-stable individuals, adjusting for age, race, sex, and study site. Older adults with one-SD higher UWL metabolite score had significantly higher mortality (HR = 1.44 [1.23, 1.52]) and mobility limitation risk (HR = 1.23 [1.15, 1.32]). The UWL metabolite score attenuated 39% of the relationship UWL and mortality, greater than 11% attenuated by traditional risk factors (i.e. demographics, lifestyle, and health conditions) and 16% by clinical biomarkers (cholesterol, glucose, kidney function, and inflammation biomarkers). Similarly, the UWL metabolite score attenuated 27% of the relationship between UWL and mobility limitation risk, contributing an additional 22% beyond traditional risk factors. This targeted metabolic characterization of UWL captures a substantial portion of the mortality and mobility limitation risks not explained by commonly measured health indicators, highlighting its potential value in risk assessment among older adults.

